# Impaired Expression of GABA Signaling Components in the Alzheimer’s Disease Middle Temporal Gyrus

**DOI:** 10.3390/ijms21228704

**Published:** 2020-11-18

**Authors:** Karan Govindpani, Clinton Turner, Henry J Waldvogel, Richard L M Faull, Andrea Kwakowsky

**Affiliations:** 1 Centre for Brain Research, Department of Anatomy and Medical Imaging, Faculty of Medical and Health Sciences, University of Auckland, Auckland 1023, New Zealand; k.govindpani@auckland.ac.nz (K.G.); ClintonT@adhb.govt.nz (C.T.); h.waldvogel@auckland.ac.nz (H.J.W.); rlm.faull@auckland.ac.nz (R.L.M.F.); 2 Department of Anatomical Pathology, LabPlus, Auckland City Hospital, Auckland 1023, New Zealand

**Keywords:** Alzheimer’s disease, GABA, GABAergic system, GABA receptors, GABA transporters, middle temporal gyrus

## Abstract

γ-aminobutyric acid (GABA) is the primary inhibitory neurotransmitter, playing a central role in the regulation of cortical excitability and the maintenance of the excitatory/inhibitory (E/I) balance. Several lines of evidence point to a remodeling of the cerebral GABAergic system in Alzheimer’s disease (AD), with past studies demonstrating alterations in GABA receptor and transporter expression, GABA synthesizing enzyme activity and focal GABA concentrations in post-mortem tissue. AD is a chronic neurodegenerative disorder with a poorly understood etiology and the temporal cortex is one of the earliest regions in the brain to be affected by AD neurodegeneration. Utilizing NanoString nCounter analysis, we demonstrate here the transcriptional downregulation of several GABA signaling components in the post-mortem human middle temporal gyrus (MTG) in AD, including the GABA_A_ receptor α_1_, α_2_, α_3_, α_5_, β_1_, β_2_, β_3_, δ, γ_2_, γ_3_, and θ subunits and the GABA_B_ receptor 2 (GABA_B_R2) subunit. In addition to this, we note the transcriptional upregulation of the betaine-GABA transporter (BGT1) and GABA transporter 2 (GAT2), and the downregulation of the 67 kDa isoform of glutamate decarboxylase (GAD_67_), the primary GABA synthesizing enzyme. The functional consequences of these changes require further investigation, but such alterations may underlie disruptions to the E/I balance that are believed to contribute to cognitive decline in AD.

## 1. Introduction

Alzheimer’s disease (AD) is a chronic neurodegenerative disorder of old age with a gradual, insidious presentation [1]. While the underlying etiology of the disease is yet to be elucidated, it is classically associated with the appearance of characteristic pathological markers including the deposition of β-amyloid (Aβ) protein as insoluble extracellular plaques and the formation of intracellular neurofibrillary tangles (NFTs) composed of hyperphosphorylated tau protein (p-tau) [2,3,4,5]. Early neuropathological changes are mostly confined to the hippocampal formation [6,7,8], with the particularly prominent loss of excitatory glutamatergic pyramidal neurons and synaptic connections in this region [9,10,11,12,13]. The progression of pathology, including the loss of excitatory connections, follows a stereotyped pattern, with secondary degeneration observed in the temporal cortex in the early stages of pathogenesis [6,14,15,16,17]. The depletion of glutamatergic connections and glutamate receptors in these regions contributes significantly to the memory and cognitive deficits characteristic of the clinical condition [18,19,20,21,22]. Normal cognitive function and memory formation within the hippocampus and cortex is reliant on the careful maintenance of a balance between excitatory and inhibitory signaling [23,24,25,26], the disruption of which may cause impairments in synaptic plasticity and network synchronization [27,28,29]. Indeed, disturbances in network synchronization and oscillatory activity have been widely reported in the AD brain [30,31,32,33]. The glutamatergic system is only one of several neurotransmitter systems involved in AD pathogenesis.

It was long thought that the inhibitory γ-aminobutyric acid (GABA) system is relatively unaffected in AD. Several studies have reported the relative preservation of GABAergic interneurons in various brain regions and their apparent resistance to Aβ-induced degeneration [34,35,36,37]. However, a large body of evidence now points to a more subtle remodeling of GABAergic circuits in the AD brain, likely progressing with disease stage [38,39,40]. At the macrostructural level, the pruning of GABAergic terminals has been reported, particularly in the vicinity of Aβ plaques, indicating a synaptic loss of function despite the preservation of GABAergic fibers [41,42]. Such changes likely underlie the pyramidal hyperexcitability observed in AD, contributing to excitatory/inhibitory (E/I) imbalance [27,38,43]. Many early studies also reported alterations in GABA levels, typically a reduction in total neurotransmitter concentration in several regions of the post-mortem AD brain [38,44]. This by itself is perhaps difficult to interpret due to the effect of agonal state changes on GABA levels in the post-mortem brain [45,46,47,48]. However, post-mortem studies have also demonstrated alterations in the subunit configurations of GABA_A_ receptors (GABA_A_Rs) in the hippocampus and cortex at the transcriptional and protein level, contributing to the remodeling of the system [48,49,50,51,52,53,54,55,56]. GABA_A_Rs are ionotropic receptors with multi-subunit structures, most commonly comprising five subunits arranged around a central Cl^-^ channel [57]. Receptors may be assembled from several potential subunit isoforms, each belonging to one of eight characterized subunit families—α, β, γ, δ, ρ, ε, θ and π [58,59]. GABA_A_R subunit isoforms are encoded by at least 21 genes, conferring this class of receptors with significant structural heterogeneity [60,61]. The precise subunit configuration of the GABA_A_R defines its pharmacological and electrical properties and the composition of post-synaptic GABA_A_Rs shapes the inhibitory post-synaptic response [58,62,63]. The restructuring of GABA_A_Rs in the AD brain likely points to a more general alteration in the function of the cerebral inhibitory system. The GABA_B_ receptor (GABA_B_R) on the other hand is a metabotropic receptor, comprising two distinct subunits termed GABA_B_ receptor 1 (GABA_B_R1) and GABA_B_ receptor 2 (GABA_B_R2). These receptors are functionally distinct from GABA_A_Rs, interacting with K^+^ channels, Ca^2+^ channels and adenylate cyclase to modulate membrane potential and couple GABA binding to downstream signaling events [64]. The downregulation of GABA_B_Rs has been previously reported in the superior frontal gyrus in the AD brain [65] and transient upregulation of GABA_B_R1 has also been observed in the AD hippocampus in the early stages of the disease [66].

GABA transporters (GATs) are involved predominantly in the removal of GABA from the synaptic cleft following neurotransmission. The four main GAT subtypes within the human brain are GABA transporter 1 (GAT1), GABA transporter 2 (GAT2), GABA transporter 3 (GAT3) and the betaine-GABA transporter (BGT1) [67]. GAT expression is likely altered in the AD brain, as reported in a few previous studies [68,69,70]. We have previously demonstrated differential alterations in GAT expression within the AD superior temporal gyrus (STG) using an immunohistochemical (IHC) approach. We noted a particularly significant upregulation in BGT1 expression concomitant with the downregulation of GAT1 in this region [70]. Expression changes in glutamate decarboxylase (GAD), the primary GABA synthesizing enzyme, have also been reported in the AD prefrontal and temporal cortex [54,71]. Taken together, these results lend further support to the idea of a general inhibitory deficit in the AD brain, affecting multiple molecular mechanisms within the GABA signaling system.

Given the likely contribution of GABAergic dysfunction to E/I imbalances in the AD hippocampus and temporal cortex, it is important to characterize the development of molecular GABAergic deficits in these brain regions. Limon et al. have previously investigated GABAergic changes in human AD temporal cortex homogenates. Using quantitative polymerase chain reaction (qPCR), this group reported the downregulation of GABA_A_R α_1_, α_2_, α_5_, β_2_, β_3_, γ_2_ and δ subunit mRNA transcripts and the preservation of GABA_A_R β_1_, γ_1_ and ρ_1_ subunit transcripts [55]. They further confirmed the downregulation of GABA_A_R α_1_ and γ_2_ subunit protein expression and the preservation of β_1_ and γ_1_ subunit protein expression using Western blot analysis [55,72]. However, an examination of subregion-dependent alterations in GABA_A_R subunit expression within the temporal cortex was not conducted. In our own subsequent IHC analysis of GABA_A_R subunit expression in the AD STG, we noted the downregulation of the α_2_ and α_5_ subunits and the preservation of the α_1_, α_3_, β_1_, β_2_, β_3_ and γ_2_ subunits [56]. These results further underscore the notion that the GABAergic system may be differentially affected in a subregion-specific manner within the AD temporal cortex.

In this study, we sought to extend our investigations into GABA receptor subunit expression changes in AD to the middle temporal gyrus (MTG), with the careful anatomical isolation of this cortical subregion and the precise quantification of mRNA transcripts utilizing NanoString nCounter analysis. We also investigated transcriptional alterations in the GABA transporters, GADs and the GABA-metabolizing enzyme aminobutyric acid transaminase (ABAT) within the AD MTG. GABA transporter expression has not previously been studied within the AD MTG. We demonstrate here the transcriptional downregulation of several GABA_A_R and GABA_B_R subunits within the MTG in the AD brain, along with the downregulation of GAD_67_ mRNA and the upregulation of GAT2 and BGT1 transcripts.

## 2. Results

**Figure 1:**
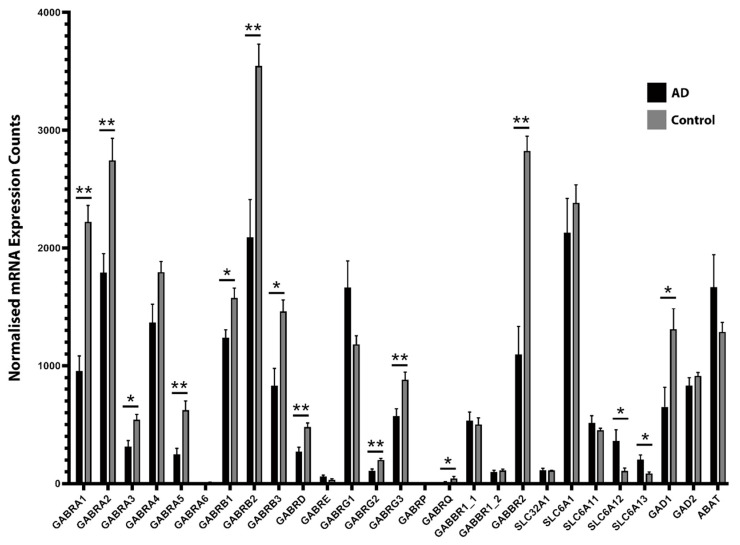
Normalized NanoString nCounter mRNA expression counts for γ-aminobutyric acid (GABA)ergic signaling components in Alzheimer’s disease (AD) and control human middle temporal gyrus (MTG) grey matter samples. Several GABAergic signaling components, including GABA_A_R subunits, the GABA_B_R2 subunit, GABA transporters, and the GABA synthesizing enzyme GAD_67_, are altered in expression in the AD MTG. Raw mRNA counts were normalized to positive control and housekeeping gene counts and background-corrected using negative control counts. For each subunit, normalized mRNA counts were compared between AD and control groups using the Mann–Whitney unpaired test, * *p* ≤ 0.05 and ** *p* ≤ 0.01. Data presented as mean ± SEM, *n* = six AD cases and six control cases. Gene names on *x*-axis; GABBR1_1 and GABBR1_2 represent two distinct transcript variants encoded by the *GABBR1* gene.

**Figure 2:**
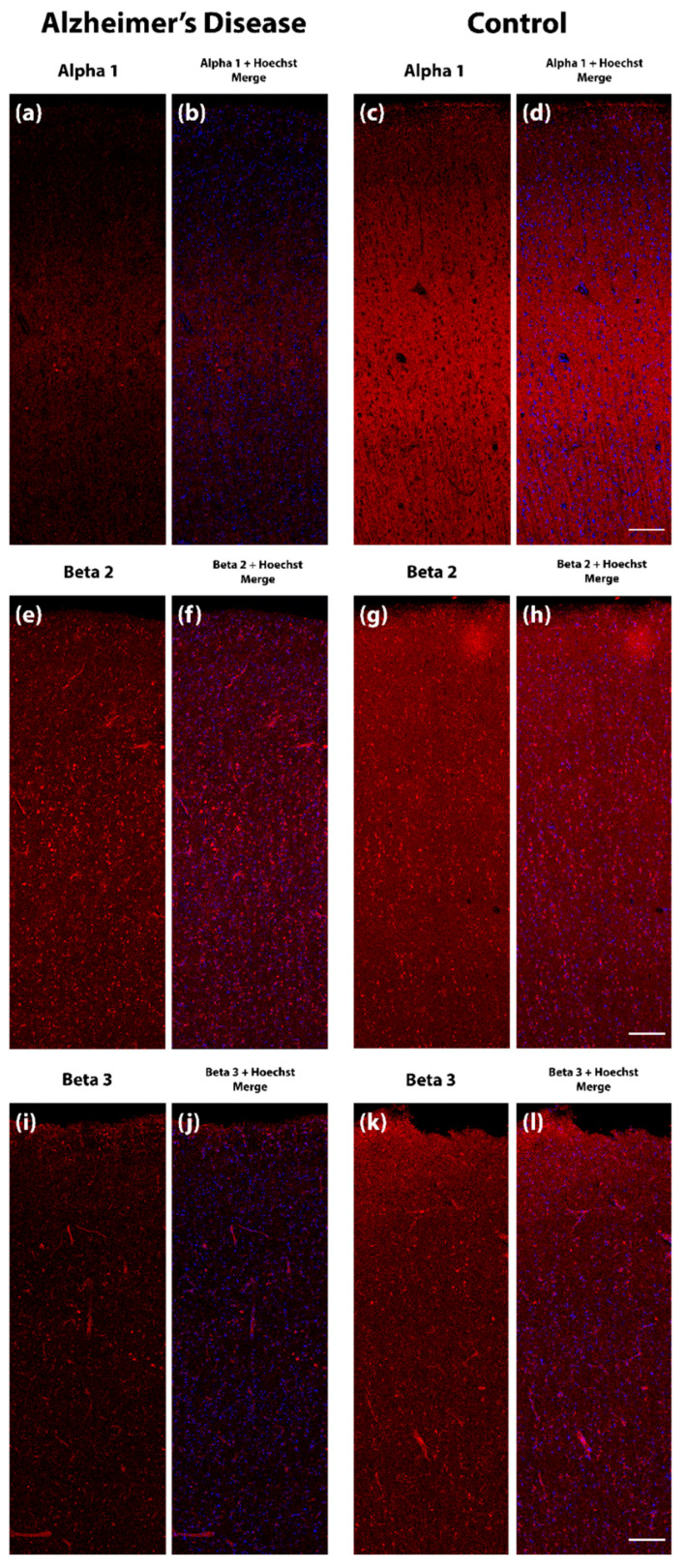
Representative fluorescent immunohistochemistry images from the human Alzheimer’s disease and control middle temporal gyrus (MTG). Post-mortem human MTG sections were labelled with antibodies against GABA_A_R subunits and imaged with uniform settings for each subunit on a confocal microscope. GABA_A_R subunits are presented in red and Hoechst staining is presented in blue. A reduction in fluorescent intensity across cortical layers was apparent for the α_1_ subunit (cases AZ90 and H122 presented) (**a**–**d**), β_2_ subunit (cases AZ90 and H122 presented) (**e**–**h**) and β_3_ subunit (cases AZ90 and H181 presented) (**i**–**l**). Scale bar, 200 µm.

NanoString nCounter analysis revealed the transcriptional downregulation of several GABAergic system components in the human AD MTG compared with age-matched control cases (Figure 1). This was particularly notable for GABA_A_R subunit transcripts, with significant transcriptional downregulation of the α_1_, α_2_, α_3_, α_5_, β_1_, β_2_, β_3_, δ, γ_2_, γ_3_, and θ subunits (encoded by the *GABRA1*, *GABRA2*, *GABRA3*, *GABRA5*, *GABRB1*, *GABRB2*, *GABRB3*, *GABRD*, *GABRG2*, *GABRG3* and *GABRQ* genes, respectively). The transcriptional expression of the GABA_A_R α_4_ subunit (*GABRA4*) was not significantly altered in the AD MTG, but there was a trend toward downregulation. A trend toward transcriptional upregulation was noted in the GABA_A_R γ_1_ subunit (*GABRG1*), but this was similarly non-significant. The expression of the GABA_A_R ε subunit (*GABRE*) was stable in AD cases. Therefore, most GABA_A_R subunits appear to be downregulated at the transcriptional level in the AD MTG. The GABA_A_R α_6_ and π subunit transcripts (*GABRA6* and *GABRP*, respectively) were not detectable above background levels in these human MTG lysates, while the ε and θ subunits were expressed at very low levels. We also noted the transcriptional downregulation of the GABA_B_R2 subunit (encoded by *GABBR2*), one of the two metabotropic receptor subunits that form the functional GABA_B_R. The expression of both the R1a and R1b splice variants of the GABA_B_R1 subunit (respectively GABBR1_1 and GABBR1_2 in Figure 1) remained unaltered. Using fluorescence immunohistochemistry (fIHC) to label formalin-fixed MTG sections from AD and control cases, we demonstrated qualitatively that marked downregulations in α_1_, β_2_ and β_3_ GABA_A_R subunits are also perceptible within the grey matter of the AD MTG at the protein level (Figure 2).

The mRNA expression of the two primary synaptic GABA transporters—GAT1 and GAT3 (encoded by *SLC6A1* and *SLC6A11*, respectively)—did not appear to be altered in the AD MTG and vesicular GABA transporter (vGAT) (*SLC32A1*) mRNA expression also remained unaltered. However, we found that both BGT1 and GAT2 transcripts (*SLC6A12* and *SLC6A13*, respectively) were upregulated.

Finally, we noted the transcriptional downregulation of the 67 kDa isoform of the GABA synthesizing enzyme glutamate decarboxylase (GAD_67_) (encoded by *GAD1*), but not the 65 kDa isoform (GAD_65_) (encoded by *GAD2*). The transcriptional expression of ABAT (encoded by *ABAT*) was also unaltered in the AD MTG.

## 3. Discussion

In this study, we have demonstrated, for the first time, the transcriptional downregulation of several GABA_A_R subunits across a defined region of the MTG in AD. In particular, the α_1_, α_2_, α_3_, α_5_, β_1_, β_2_, β_3_, δ, γ_2_, γ_3_, and θ subunits were downregulated in tissue samples from the AD MTG, while the α_4_, γ_1_ and ε subunits were preserved. Using fIHC, we demonstrated qualitatively the downregulation of GABA_A_R α_1_, β_2_ and β_3_ subunits in AD MTG sections, indicating the possibility of a concomitant downregulation in these subunit proteins. While this could indicate a remodeling of GABA_A_R subunit configurations in the MTG, it may alternatively be indicative of a general downregulation in GABA_A_Rs or the loss of GABA_A_R-expressing cells in the MTG in the later stages of AD, given the fact that almost all detectable GABA_A_R subunits appear to be affected in the same manner. Indeed, given the widespread loss of glutamatergic fibers and terminals within the AD temporal cortex, along with GABAergic terminals [6,14,15,16,17,41,42], it is to be expected that there will be a concomitant reduction in GABA_A_R expression detectable in any given mass of tissue homogenate (or equally in any given volume of total RNA). However, there were some GABA_A_R subunits that did not appear to be significantly downregulated, namely the α_4_, γ_1_ and ε subunits. Indeed, there was a non-significant trend toward the transcriptional upregulation of the γ_1_ subunit. This would logically imply that even if there is some contribution of glutamatergic and GABAergic terminal loss to the overall downregulation that we observe in most GABA_A_R subunits, there is a mechanism at play whereby some GABA_A_R subunits are spared whereas others are more selectively downregulated. Within the limits of the current study, we cannot say with certainty what mechanism may underlie the relative sparing of some subunits. Indeed, this may be a transcriptional mechanism, or it could simply indicate the relative sparing of a subpopulation of neurons or other cell types expressing specific GABA_A_R variants. Given the high expression of the α_4_ and ε subunits at extrasynaptic sites, it is also conceivable that these are relatively spared during the targeted degeneration of neuronal terminals. However other prominently extrasynaptic subunits such as α_5_ and δ [73] were found to be transcriptionally downregulated in the AD MTG and may perhaps be lost with the degeneration of glutamatergic cell bodies. It is important to note that previous IHC studies have demonstrated GABA_A_R subunit downregulation in cortical and hippocampal AD tissue at levels greater than may be explainable purely through cellular degeneration, potentially indicating that there is a transcriptional deficit at play. Mizukami et al. demonstrated using *in situ* hybridization the downregulation of β_3_ subunit mRNA even in regions of the AD hippocampus exhibiting little to no cell loss, while β_2_ subunit mRNA was preserved even in regions exhibiting severe pathological changes [53]. Iwakiri et al. actually observed that γ_1/3_ subunits (using an antibody recognizing both subunits) were upregulated in the CA1–4 hippocampus and dentate gyrus in AD [48]. We too observed a non-significant trend toward upregulation of the γ_1_ subunit transcript in the AD MTG. The transcriptional upregulation of some subunits combined with neuronal and synaptosomal loss may result in the detection of overall stable expression in a given subregion. The sensitivity of any particular GABA_A_R subunit to loss within a fixed amount of tissue (or total RNA) is thus likely to be dependent on several factors, including alterations in transcription and protein trafficking, the precise localization of receptors at pre- or post-synaptic terminals or extrasynaptic sites, neuronal subtype-specific expression, and local patterns of glutamatergic and GABAergic neuronal and synaptosomal degeneration. The precise mechanisms underlying these effects remain to be determined, but our results indicate a remodeling of the overall GABA_A_R subunit composition in the AD MTG. This points to the alteration of the functional and pharmacological properties of the inhibitory system at large, whether these alterations are primary or secondary to degeneration.

While this is the first study to examine such alterations specifically in the MTG, Limon et al. have previously reported AD-associated GABA_A_R subunit expression changes across the temporal cortex [55]. Interestingly, they too reported a trend toward the transcriptional downregulation of several subunits, including α_1_, α_2_, α_5_, β_2_, β_3_, γ_2_ and δ. This supports our observation that the same subunit mRNAs are downregulated within the MTG in particular. Their study, like ours, also noted the sparing of γ_1_ and ε subunit mRNA expression, indicating that this is likely uniform across the temporal cortex. However, this group reported the sparing of β_1_ and θ subunit mRNA expression, while we noted a downregulation in both. This likely indicates a specific loss of these subunits within the MTG in AD, while this may not be the case across the temporal cortex as a whole. It should also be noted that NanoString nCounter analysis is a more sensitive method of transcript quantification than qPCR, giving us a greater ability to detect more subtle alterations in transcript expression. Howell et al. have reported the preservation of GABA_A_R α_5_ subunit expression in the AD temporal cortex as a whole, however they had quantified subunit protein expression using autoradiography [74], a generally low-resolution technique. As stated earlier, we have previously observed the downregulation of the GABA_A_R α_2_ and α_5_ subunits in the AD STG, with the preservation of α_1_, α_3_, β_1_, β_2_, β_3_ and γ_2_ subunits [56]. There may therefore be differences in the sensitivity of GABA_A_R subunits to AD-associated expression changes within the MTG and STG, pointing to differences in subregional vulnerability within the temporal cortex.

We also observed the transcriptional downregulation of the GABA_B_R2 subunit in the AD MTG, while the GABA_B_R1 subunit appeared to be spared. This is the first study to examine alterations in GABA_B_R subunit expression within the human temporal cortex. It is traditionally considered that the formation of a functional GABA_B_R necessitates the association of both the GABA_B_R1 and GABA_B_R2 subunits, given the presence of an endoplasmic reticulum (ER) retention signal on the GABA_B_R1 subunit that is masked by association with GABA_B_R2. The GABA_B_R2 subunit, on the other hand, contains the orthosteric binding site for GABA [75]. The specific downregulation of the GABA_B_R2 subunit may indicate the downregulation of surface-expressed GABA_B_Rs, likely resulting in a reduction in functional inhibition in GABA_B_R-expressing cells. Given the preservation of GABA_B_R1 subunit transcripts, there may well be increased ER retention of GABA_B_R1. There is evidence that GABA_B_R1 subunits in the ER membrane may have alternative roles, participating in intracellular signaling pathways including the ERK/MAPK pathway [76,77], potentially explaining the differential transcriptional changes in these two metabotropic receptor subunits in the AD MTG. Considering the stability of GABA_B_R1 expression compared with GABA_B_R2 expression, it is unlikely that the measured reduction in the latter is purely the result of neuronal or synaptosomal loss.

The functional implication of such a general transcriptional down-regulation in both GABA_A_R and GABA_B_R subunits is difficult to determine in isolation. However, these results support the idea that AD is associated with the widespread disruption of functional inhibition in brain, including in the temporal cortex. The remodeling of GABA_A_R subunit configurations on surviving excitatory neurons in the middle to late stages of AD may have subtle but significant implications for the precise pharmacological control of neuronal inhibition, altering GABA_A_R channel opening and gating, sensitivity to GABA and other endogenous modulators, and GABA_B_R coupling to downstream signaling pathways and membrane-bound ion channels. Within any given cortical region, this could have a potentially profound impact on the balance between excitation and inhibition in surviving neuronal networks. Therefore, our understanding of E/I balance disruption in the AD temporal cortex requires a factoring of GABAergic remodeling in addition to the well-characterized glutamatergic degeneration.

In the AD MTG, we did not detect transcriptional changes in the primary synaptic GABA transporters—GAT1 and GAT3. This may indicate the general preservation of GABA clearance mechanisms at the synapse. Two previous studies have investigated temporal cortex GAT1/3 expression using radioligand binding techniques. Simpson et al. reported the loss of binding sites for ^3^[H]nipecotic acid, a non-specific inhibitor of all GABA transporters except BGT1, in AD temporal cortex samples [68], while Nägga et al. reported the preservation of binding sites for ^3^[H]tiagabine, a specific inhibitor of GAT1 [69]. However, neither study segregated their data by temporal cortex subregion. Our group has previously used IHC techniques to demonstrate the downregulation of GAT1 in the AD STG, with the sparing of GAT3 expression [70]. The current study is, however, the first to examine alterations in GAT1 and GAT3 mRNA expression specifically in the AD MTG, where these species appear to be well conserved. More interesting was our observation of transcriptional upregulation of BGT1 and GAT2 in this region. We have previously reported the same for BGT1 in the STG using IHC, indicating the potential for a general BGT1 upregulation across the temporal cortex in AD. This is perhaps not altogether surprising. BGT1 is not believed to play a significant role in synaptic GABA uptake, being a low-affinity GABA transporter with relatively slow transport kinetics compared with GAT1/3. However, several recent studies have demonstrated a novel role for astrocytic BGT1 in cytoprotective mechanisms, with significant BGT1 upregulation noted in response to osmotic stress and kainate-induced injury [78,79,80]. We have ourselves previously demonstrated the upregulation of BGT1 in astrocytes within the STG in AD [70]. Therefore, the transcriptional upregulation of BGT1 that we observe here may potentially represent a similar astrocytic upregulation. The transcriptional upregulation of GAT2 is more cryptic, as the precise physiological role of this transporter in the human brain is unknown. However, GAT2 is reportedly expressed on a subpopulation of blood vessels and may play a role in the maintenance of cortical osmolarity through the transport of the osmolyte taurine out of the brain [81].

Finally, we observed in the AD MTG the transcriptional downregulation of the GAD_67_ isoform of GAD, the GABA synthesizing enzyme, but not the GAD_65_ isoform. Schwab et al. previously reported the opposite expressional pattern in the MTG at the protein level—The downregulation of GAD_65_ expression, as measured by IHC and Western blot analysis, and the preservation of GAD_67_ expression, as measured by Western blot analysis [71]. It should be noted however that their Western blot study employed only two AD and two control cases, so it is difficult to draw firm conclusions on this basis. Reinikainen et al. reported in an early study that GAD activity is preserved in human AD temporal cortex homogenates, suggesting that GAD levels across the temporal cortex are not altered [82]. The transcriptional downregulation of GAD_67_ that we observe in the AD MTG may be specific to this subregion of the temporal cortex. This may account for the numerous reports in the literature of reduced GABA neurotransmitter concentrations in the temporal cortex [36,45,83,84,85,86] as the GAD_67_ isoform is predominantly responsible for the synthesis of GABA under basal conditions and accounts for the majority of intracellular cytosolic GABA [87,88]. GAD_65_ is targeted to synaptic terminals and plays a greater role in the synaptic release of GABA [89]. The ABAT enzyme, responsible for GABA catabolism, appears to be transcriptionally preserved in the AD MTG, and in association with GAT1/3 preservation, this may indicate that GABA removal and recycling mechanisms at the synapse are relatively spared in the AD MTG.

The precise effect of these alterations in GABAergic signaling component expression remains to be determined. However, it may be hypothesized that the subregion-specificity of such changes results in differential effects on inhibition in different regions of the temporal cortex at any given stage of AD pathogenesis. This may naturally imply the evolution of subregion-specific differences in the firing characteristics of excitatory neurons in the temporal cortex, dependent on the extent and nature of GABA_A_R subunit remodeling. Equally, our observation of a general downregulation in most GABA_A_R subunit transcripts and the GABA_B_R1 subunit in the MTG may indicate a more general reduction in GABAergic inhibitory control, conceivably contributing to glutamatergic hyperexcitability in this subregion. Synaptosomal degeneration at inhibitory synapses, coupled with synaptic GABA receptor loss and/or remodeling, could have a profound effect on overall network characteristics. The precise subregion-specific characterization of molecular changes in the inhibitory system therefore contributes to our understanding of the anatomical evolution of these deficits during AD pathogenesis.

In closing, it is perhaps worth considering the implications of such a widespread GABA_A_R remodeling to AD symptomatology and therapy. In the introduction, we briefly mentioned mechanisms that may underlie alterations in pyramidal excitability, including inhibitory synaptosomal loss [41,42] and alterations in tonic GABA_A_R-mediated inhibition [90,91]. Such alterations are likely to play a significant role in the cognitive and memory deficits inherent in the disease. However, other signs of this E/I dysregulation are also apparent in AD, particularly the high incidence of epileptiform activity, including non-motor seizures in the advanced disease [92]. It is interesting to note that temporal lobe epilepsy (TLE) is also associated with alterations in GABA_A_R receptor configurations in the temporal cortex and hippocampus [93,94,95,96]. While it is difficult to draw direct parallels between GABA_A_R receptor plasticity in the two conditions, it is plausible that subunit remodeling has similar functional consequences in the AD temporal cortex alongside other complex Aβ-induced alterations in inhibitory signaling. Alterations in GABA_A_R subunit configurations could also alter the pharmacology of GABAergic drugs prescribed to patients with AD, potentially for the treatment of co-morbid conditions. Indeed, benzodiazepines (BZDs), a common class of anxiolytic drugs targeting γ_2_ subunit-containing GABA_A_Rs, are commonly prescribed to AD patients for the treatment of behavioral and psychological symptoms [97]. Subunit remodeling and changes in receptor pharmacology could have implications for BZD efficacy in AD patients, but this has not been adequately studied. Prolonged use of BZDs may itself be associated with increased risk of AD in elderly patients [98] and it is not clear how GABAergic-targeted therapies may affect the molecular evolution of inhibitory deficits in the AD brain. Specific GABA_A_R subtypes are now emerging as promising targets in the treatment of cognitive deficits and neuronal loss in AD, including the extrasynaptic α_5_ subunit-containing receptor [99,100,101,102]. Therefore, studies such as ours, elucidating subregional GABA_A_R subunit alterations in AD, could improve the potential for therapeutic targeting in the future.

## 4. Materials and Methods

### 4.1. Post-Mortem Human Brain Tissue Processing

Human brain tissue was obtained with informed consent through a donor program at the Neurological Foundation Human Brain Bank at the University of Auckland. All work with human tissue was conducted with the prior approval of the University of Auckland Human Participants Ethics Committee. Tissue from all human cases used in this study had previously been assessed by a neuropathologist and detailed case histories were taken at the time of donation. Control cases were confirmed to have no history of neurological impairment or evidence of neurodegenerative disorders. The control cases did not display tau or amyloid pathology and all AD cases were classified as Braak stage IV-VI based on NFT pathology [6]. In addition, we only considered AD cases that were categorized as Probable AD or Definite AD, based on the criteria established by the Consortium to Establish a Registry for Alzheimer’s Disease (CERAD) [103]. In every case, control or AD, the cause of death was unrelated to brain trauma or neurological disease aside from AD dementia for the relevant disease cases. As far as possible, AD and control cases were matched for age, sex and post-mortem delay. Details of the cases used are presented in Table 1, Table 2, Table 3 and Table 4. All tissue was processed following protocols that had been developed and validated previously by our group. In brief, the brain is separated into left and right hemispheres, with the right hemisphere perfused with formalin before freezing and the left hemisphere frozen fresh. Each hemisphere is dissected according to a physical–functional map of the brain, resulting in a series of blocks that reproduce the sub-regional organization of several cortical gyri and subcortical structures. This makes it possible to precisely define a subregion of interest for anatomical or molecular investigation. The full procedure for dissection is provided by Waldvogel, et al. [104].

Human brains were transported to the Neurological Foundation Human Brain Bank with minimal post-mortem delay and the left and right hemispheres separated. Once the hemispheres of the brain are separated, the left hemisphere is immediately processed by dissecting into physical–functional blocks. The blocks are flash-frozen with powdered dry ice and stored at −80 °C. These may later be sectioned with a cryostat as required. The blocks to be used were later cut into 60 µm-thick sections using a cryostat and the cortical grey matter was isolated carefully with the use of a scalpel. These grey matter samples were frozen at −80 °C for later RNA isolation. In this manner, MTG tissue from 6 AD and 6 age-matched control cases was collected (Table 1 and Table 2).

For free-floating IHC, formalin-fixed tissue was used. After separation, the right hemisphere of the brain was flushed through the carotid, vertebral and anterior cerebral arteries with pH 7.4 phosphate-buffered saline (PBS) containing 1% sodium nitrite, to clear the tissue of blood and induce mild vasodilation. The hemisphere was then perfused in the same manner for 30–45 min with 15% formalin in 0.1 M phosphate buffer, pH 7.4. Following perfusion–fixation, the whole hemisphere was post-fixed by immersing in the same 15% formalin solution for 6–12 h at room temperature (RT). The tissue was then ready for dissection into blocks. The blocks themselves were immersed in fresh fixative for 24–48 h, transferred into a solution of 20% sucrose in 0.1 M phosphate buffer with 0.1% sodium azide for 2–3 days, and then transferred into a solution of 30% sucrose in 0.1 M phosphate buffer with 0.1% sodium azide for another 2–3 days to cryopreserve the tissue. Finally, the formalin-fixed blocks were snap-frozen on powdered dry ice and stored at −80 °C. The required blocks were later cut into 50 µm sections at −35 °C using the HM 450 sliding microtome (Thermo Fisher, Waltham, MA, USA), and these sections were stored at 4 °C in PBS with 0.1% sodium azide.

### 4.2. TRIzol RNA Isolation from Fresh-Frozen Human MTG Tissue

RNA isolation from post-mortem tissue was conducted using the RNeasy Lipid Mini Kit (Qiagen, Hilden, Germany, Cat. 74804) using the kit protocol. Tissue samples on dry ice were treated with 1 mL of the QIAzol Lysis Reagent and immediately ground with the use of a pestle followed by syringe homogenization (25-gauge needle). The resulting homogenates were incubated in QIAzol for 5 min at room temperature (RT), after which chloroform was spiked into the homogenate (0.2 mL for every 1 mL of QIAzol). The tubes containing the tissue–QIAzol–chloroform mixture were shaken vigorously for 15 s and incubated for 3 min at RT. Phase separation was achieved by centrifugation at 15,000× *g* for 15 min at 4 °C. For each sample, the aqueous phase was carefully separated with a pipette and transferred to a fresh Eppendorf and one volume of ice-cold 70% ethanol was added to each sample. After brief mixing, the mixtures were transferred into RNeasy spin columns and centrifuged at 8000× *g* for 15 s. In total, 700 µL of Buffer RW1 was added to each column and they were centrifuged at 8000× *g* for a further 15 s. In total, 500 µL of Buffer RPE was washed through the column by centrifuging at 8000× *g* for 15 s, and this was repeated a second time. The spin columns were dried by centrifuging at 11,500× *g* for 1 min, and then RNA was eluted by adding RNase-free water to the spin columns and centrifuging at 8000× *g* for 1 min. RNA sample purity was assessed using the NanoDrop spectrophotometer (Thermo Fisher, Waltham, MA, USA) and RNA integrity was tested with the Agilent 2100 Bioanalyzer (Agilent Technologies, Santa Clara, CA, USA). In all cases, samples displayed DV300 ≥ 60. RIN values ranged from ~5–7 for control tissue and ~4–6 for AD tissue.

### 4.3. RNA Quantification by NanoString nCounter Analysis

The NanoString nCounter assay was outsourced to The Otago Genomics Facility (University of Otago, Dunedin, New Zealand), and assays were performed as described by Geiss et al. [105]. An nCounter Custom CodeSet library of RNA-specific probes were designed to target transcripts encoded by 26 human genes involved in cerebral GABAergic function and a further 4 housekeeping genes (ACTB, B2M, GAPDH and TOP1) (Table 5). The Custom CodeSet library included both a Capture and Reporter CodeSet specific for each gene of interest. For each gene, probes were designed to hybridize with all known transcript variants, except for *GABBR1*, for which probes were designed to differentially target the R1a and R1b transcript variants.

Briefly, the assay was performed with 100 ng of total RNA per tissue sample. In total, 100 ng total RNA in a 5 µL volume of water was mixed with 3 µL of Reporter CodeSet, 2 µL of Capture CodeSet and 5 µL of hybridization buffer. The hybridization mixture was incubated at 65 °C for 21 h in a thermal cycler. Samples were then loaded onto nCounter Prep Plates for purification before being loaded into nCounter Cartridges for immobilization. Cartridges were quantified using the GEN2 Digital Analyzer (NanoString Technologies, Seattle, WA, USA), a multi-channel epifluorescence scanner for nCounter cartridges, and the “max” field-of-view option selected (555 images per sample). Reporter signals were thus counted across all 555 images. Raw counts per lane were saved to a Reporter Code Count (RCC) file.

Statistical analysis and quality control were conducted using the nSolver Software v.4 (NanoString Technologies, Seattle, WA, USA). A Reporter Library File (RLF) specifying the identities of reporter probe signals was loaded into nSolver, along with the RCC file. Prior to normalization, the mean of raw negative control counts for each sample (8 internal negative control RNA sequences) was subtracted from target counts using nSolver; this was done to prevent comparisons being made for targets that were not actually expressed above background levels in the MTG. Raw counts for all samples and targets were normalized to the geometric mean of averaged raw counts for the 6 positive controls across the same samples. CodeSet content normalization was conducted to determine sample-specific correction factors for all counts for each individual sample; this is based on a normalization to the geometric mean of housekeeping gene average raw counts for each sample. This normalization procedure produces a single compound normalization factor for counts from each sample, allowing raw data to be corrected for technical variability in assay procedure. In addition, for each sample, the raw counts of the 8 internal negative control RNA sequences were manually normalized using the same compound normalization factor that had been applied by nSolver to target raw counts for that sample.

To determine whether normalized mRNA counts for each target differed significantly between AD and control MTG tissue, normalized counts from 6 AD and 6 control cases were compared using the Mann–Whitney unpaired test. RNA target expression was considered to be altered if there was a statistically significant difference between normalized counts for each group (*p* ≤ 0.05). A non-parametric test was used as data did not meet the assumptions of a parametric test, i.e., normality and homogeneity of variance.

### 4.4. Free-Floating Fluorescence Immunohistochemistry (fIHC)

Seven control and 7 AD cases were stained to assess GABA_A_R subunit expression (Table 3 and Table 4). Two MTG sections per case were washed overnight with PBS supplemented with 0.2% Triton X-100 (PBST). Following this, sections were washed with PBST a further three times for 10 min and treated with primary antibodies raised against the GABA_A_R α_1_ subunit (mouse monoclonal, clone BD24, gifted by Jean-Marc Fritschy, Switzerland; 1:5000), β_2_ subunit (rabbit polyclonal, Novus, NBP1-51214; 1:1000) or β_3_ subunit (mouse monoclonal, Novus, NBP1-47613; 1:1000) for 72 h at 4 °C. All antibodies were diluted in an immunobuffer consisting of 1% normal goat serum and 0.04% *w*/*v* merthiolate in PBST. Sections were washed with PBST three times for 10 min and then treated with the appropriate Alexa Fluor goat secondary antibodies (Anti-Rabbit IgG Fluor 647, Thermo Fisher, A21245; Anti-Mouse IgG Alexa Fluor 647, Thermo Fisher, A21236) diluted 1:500 in immunobuffer for 24 h at RT. Sections were washed once with PBST and treated with Hoechst 33,342 (Molecular Probes, H-3570; 1:1000). Sections were washed three times in PBS and were then mounted onto glass microscopy slides with Mowiol-488 mounting medium and coverslipped with #1.5 glass coverslides. Fluorescent imaging was conducted using the LSM710 laser-scanning inverted confocal microscope (Carl Zeiss AG, Oberkochen, Germany). A 2 × 6 tile-scan image was taken for each section encompassing all layers of the cortex. AD and control sections stained for each GABA_A_R subunit were imaged at the same settings to ensure comparability and analysed qualitatively.

## Figures and Tables

**Table 1:** Details of control cases for NanoString nCounter Study.

Case	Age	Sex	PM Delay (h)	Cause of Death	Brain Weight (g)
H122	72	F	9	Emphysema	1230
H123	78	M	7.5	Abdominal aortic aneurysm	1260
H131	73	F	13	Ischaemic heart disease	1210
H164	73	M	13	Ischaemic heart disease	1315
H190	72	F	19	Ruptured myocardial infarction	1264
H202	83	M	14	Abdominal aortic aneurysm	1245

**Table 2:** Details of Alzheimer’s disease cases for NanoString nCounter Study.

Case	Age	Sex	PM Delay (h)	Cause of Death	CERAD Class.	Braak Stage Score	Brain Weight (g)
AZ38	80	M	5.5	Pneumonia emphysema	Definite AD	V	1039
AZ45	82	M	4.5	Pneumonia	Probable AD	IV	1200
AZ61	87	F	7.5	Dementia	Definite AD	V	1036
AZ72	70	F	7	Lung cancer	Probable AD	V	1044
AZ90	73	M	4	Gastrointestinal haemorrhage	Definite AD	V	1287
AZ96	74	F	8.5	Metastatic cancer, likely gastric	Definite AD	V	1062

**Table 3:** Details of control cases for fluorescence immunohistochemistry study.

Case	Age	Sex	PM Delay (h)	Cause of Death	Brain Weight (g)
H122	72	F	9	Emphysema	1230
H123	78	M	7.5	Abdominal aortic aneurysm	1260
H137	77	F	12	Coronary arteriosclerosis	1227
H169	81	M	24	Asphyxia	1225
H180	73	M	33	Ischemic heart disease	1318
H181	78	F	20	Aortic aneurism	1292
H202	83	M	14	Abdominal aortic aneurysm	1245

**Table 4:** Details of Alzheimer’s disease cases for fluorescence immunohistochemistry study.

Case	Age	Sex	PM Delay (h)	Cause of Death	CERAD Class.	Braak Stage Score	Brain Weight (g)
AZ38	80	M	5.5	Pneumonia emphysema	Definite AD	V	1039
AZ45	82	M	4.5	Pneumonia	Probable AD	IV	1200
AZ90	73	M	4	Gastrointestinal haemorrhage	Definite AD	V	1287
AZ92	93	F	11.5	Bronchopneumonia	Probable AD	IV	1123
AZ98	91	F	20.5	Alzheimer’s disease/atrial fibrillation	Definite AD	VI	958
AZ102	84	F	14.5	Lower respiratory tract infection	Definite AD	VI	1088
AZ103	87	M	<24	Alzheimer’s dementia	Definite AD	IV	1385

**Table 5:** Complete list of genes, isoform accession numbers and target sequences for NanoString nCounter probes.

HUGO Gene	Gene Name	Accession Number(s)
*ABAT*	GABA transaminase	NM_001127448.1; NM_020686.5; NM_000663.4
*ACTB* *	Beta-actin	NM_001101.3
*B2M* *	Beta-2-microglobulin	NM_004048.2
*GABBR1*	GABA_B_ receptor 1 subunit, isoform a	NM_001470.3
*GABBR1*	GABA_B_ receptor 1 subunit, isoform b	NM_021903.2
*GABBR2*	GABA_B_ receptor 2 subunit	NM_005458.7
*GABRA1*	GABA_A_ receptor α_1_ subunit	NM_001127643.1; NM_000806.5; NM_001127648.1; NM_001127645.1; NM_001127644.1
*GABRA2*	GABA_A_ receptor α_2_ subunit	NM_001330690.1; NM_001114175.2; NM_001286827.2; NM_000807.3
*GABRA3*	GABA_A_ receptor α_3_ subunit	NM_000808.3
*GABRA4*	GABA_A_ receptor α_4_ subunit	NM_001204266.1; NM_001204267.1; NM_000809.3
*GABRA5*	GABA_A_ receptor α_5_ subunit	NM_000810.3; NM_001165037.1
*GABRA6*	GABA_A_ receptor α_6_ subunit	NM_000811.2
*GABRB1*	GABA_A_ receptor β_1_ subunit	NM_000812.3
*GABRB2*	GABA_A_ receptor β_2_ subunit	NM_021911.2; NM_000813.2
*GABRB3*	GABA_A_ receptor β_3_ subunit	NM_001278631.1; NM_000814.5; NM_001191320.1; NM_021912.4; NM_001191321.2
*GABRD*	GABA_A_ receptor δ subunit	NM_000815.4
*GABRE*	GABA_A_ receptor ε subunit	NM_004961.3
*GABRG1*	GABA_A_ receptor γ_1_ subunit	NM_173536.3
*GABRG2*	GABA_A_ receptor γ_2_ subunit	NM_198903.2; NM_000816.3; NM_198904.2
*GABRG3*	GABA_A_ receptor γ_3_ subunit	NM_033223.4; NM_001270873.1
*GABRP*	GABA_A_ receptor π subunit	NM_001291985.1; NM_014211.2
*GABRQ*	GABA_A_ receptor θ subunit	NM_018558.3
*GAD1*	Glutamic acid decarboxylase, 67 kDa isoform	NM_000817.2
*GAD2*	Glutamic acid decarboxylase, 65 kDa isoform	NM_000818.2; NM_001134366.1
*GAPDH* *	Glyceraldehyde-3-phosphate dehydrogenase	NM_001256799.2; NM_001289746.1; NM_001289745.1; NM_002046.5
*SLC32A1*	Vesicular GABA transporter (vGAT)	NM_080552.2
*SLC6A1*	GABA transporter 1 (GAT1)	NM_003042.3
*SLC6A11*	GABA transporter (GAT3)	NM_001317406.1; NM_014229.2
*SLC6A12*	Betaine transporter 1 (BGT1)	NM_001206931.1; NM_003044.4; NM_001122847.2; NM_001122848.2
*SLC6A13*	GABA transporter 2 (GAT2)	NM_001190997.2; NM_001243392.1; NM_016615.4
*TOP1* *	DNA topoisomerase 1	NM_003286.3

* Housekeeping genes.

## Abbreviations

Aβ	Amyloid-beta
ABAT	4-aminobutyrate aminotransferase
AD	Alzheimer’s disease
BGT1	Betaine-GABA transporter 1
BZD	Benzodiazepine
CA	Cornu Ammonis
CERAD	Consortium to Establish a Registry for Alzheimer’s Disease
E/I	Excitatory/Inhibitory
ER	Endoplasmic reticulum
fIHC	Fluorescence immunohistochemistry
GABA	Gamma-aminobutyric acid
GABA_A_R	GABA_A_ receptor
GABA_B_R	GABA_B_ receptor
GABA_B_R1	GABA_B_ receptor subunit 1
GABA_B_R2	GABA_B_ receptor subunit 2
GAD	Glutamate decarboxylase
GAD_65_	Glutamate decarboxylase, 65 kDa isoform
GAD_67_	Glutamate decarboxylase, 67 kDa isoform
GAT	GABA transporter
GAT1	GABA transporter 1
GAT2	GABA transporter 2
GAT3	GABA transporter 3
IgG	Immunoglobulin G
IHC	Immunohistochemistry
MTG	Middle temporal gyrus
NFT	Neurofibrillary tangle
PBS	Phosphate-buffered saline
PM	Post-mortem
p-tau	Phosphorylated tau protein
qPCR	Quantitative polymerase chain reaction
RCC	Reporter code count
RLF	Reporter library file
RNA	Ribonucleic acid
RT	Room temperature
STG	Superior temporal gyrus
TLE	Temporal lobe epilepsy
vGAT	Vesicular GABA transporter
